# A good night’s sleep and the habit of net use: perceptions of risk and reasons for bed net use in Bukoba and Zanzibar

**DOI:** 10.1186/1475-2875-12-203

**Published:** 2013-06-13

**Authors:** Hannah M Koenker, Dana Loll, Datius Rweyemamu, Abdullah S Ali

**Affiliations:** 1 Johns Hopkins Bloomberg School of Public Health Center for Communication Programs, Baltimore, MD, USA; 2 Department of Sociology, University of Dar es Salaam, Dar es Salaam, Tanzania; 3 Zanzibar Malaria Control Programme (ZMCP), Ministry of Health, Zanzibar, Tanzania

**Keywords:** Malaria, Tanzania, LLIN, Bed nets, Zanzibar, Risk perception, Behaviour change communication, Qualitative

## Abstract

**Background:**

Intensive malaria control interventions in the United Republic of Tanzania have contributed to reductions in malaria prevalence. Given that malaria control remains reliant upon continued use of long-lasting insecticidal bed nets (LLINs) even when the threat of malaria has been reduced, this qualitative study sought to understand how changes in perceived risk influence LLIN usage, and to explore in more detail the benefits of net use that are unrelated to malaria.

**Methods:**

Eleven focus group discussions were conducted in Bukoba Rural district and in Zanzibar Urban West district in late 2011. Participants were males aged 18 and over, females between the ages of 18 and 49, and females at least 50 years old.

**Results:**

The perceived risk of malaria had decreased among the respondents, and malaria control interventions were credited for the decline. Participants cited reductions in both the severity of malaria and in their perceived susceptibility to malaria. However, malaria was still considered a significant threat. Participants’ conceptualization of risk appeared to be an important consideration for net use. At the same time, comfort and aspects of comfort (getting a good night’s sleep, avoiding biting pests) appeared to play a large role in personal decisions to use nets consistently or not. Barriers to comfort (feeling uncomfortable or trapped; perceived difficulty breathing, or itching/rashes) were frequently cited as reasons not to use a net consistently. While it was apparent that participants acknowledged the malaria-prevention benefits of net use, the exploration of the risk and comfort determinants of net use provides a richer understanding of net use behaviours, particularly in a setting where transmission has fallen and yet consistent net use is still crucial to maintaining those gains.

**Conclusion:**

Future behaviour change communication campaigns should capitalize on the non-malaria benefits of net use that provide a long-term rationale for consistent use even when the immediate threat of malaria transmission has been reduced.

## Background

The recent scale-up of malaria control interventions in Tanzania has dramatically reduced the risk of malaria in many regions. Given that malaria control is reliant upon continued use of long-lasting, insecticidal bed nets (LLINs) even when the threat of malaria has been reduced, this study aimed to explore factors at play when perceived threat is low, and how net use might be promoted when fear appeals are no longer effective. Through the use of qualitative methods, this study sought to understand how changes in perceived risk influence LLIN usage, and to explore in more detail the benefits of net use that are unrelated to malaria.

Between 2008 and 2011, mainland Tanzania experienced a significant reduction in malaria parasitaemia due in large part to a comprehensive and well-implemented malaria control programme. The National Malaria Control Programme (NMCP) conducted two mass campaigns with LLINs, first targeting children under five [1] and then the general population [2,3], conducted indoor residual spraying (IRS), scaled-up access to artemisinin combination therapy (ACT) and intermittent preventive treatment for pregnant women (IPTp) and carried out large scale, social and behaviour change communication efforts [4]. Zanzibar’s parasitaemia rates have fallen significantly since 2004, thanks to the introduction of ACT in 2003, ad-hoc LLIN campaigns in 2005–6 and 2008, and yearly IRS since 2006 [5,6]. The recent Malaria Indicator Surveys showed that in Zanzibar, malaria prevalence stood at 0.8% in 2007 and remained less than 1.0% in the 2011–12 THMIS. In Kagera Region, prevalence fell from 41.1% in 2007–8 to 8.5% in 2011–12. LLIN ownership in both areas was high with use rates lower in Zanzibar (Table 1) [7].

**Table 1:** Long-lasting insecticidal bed net ownership and use in study sites from 2011–2012 Tanzania HIV/AIDS and Malaria Indicator Survey

	**Ownership of at least one net/LLIN**	**Average number of nets per household (any net/LLIN)**	**Use of net/LLIN (general population)**	**Use of LLIN among households with at least 1 LLIN**
Kagera Region (n = 445)	94.2%/91.9%	2.3/2.1	70.7% / 66.9%	72.6%
Zanzibar (Unguja) (n = 141)	84.3%/65.5%	2.3/1.6	57.6% / 36.7%	58.6%

Note: Fieldwork for the 2011–2012 THMIS was done concurrently with the fieldwork for this study.

The use of nets is driven by a variety of factors, including first and foremost the availability of nets within the household [8], and then by mosquito density, seasonality, risk perception, social factors, and practical issues [9-17]. Non-use of nets has been most often reported to be due to perceived low mosquito density and to discomfort, generally due to heat [16]. Net use messaging in Tanzania and in many other countries has focused almost entirely on the prevention of malaria as the key reason to use nets. Key messages that have focused on the threat of the disease may no longer resonate with populations who do not perceive malaria to be a significant danger. If prevention of malaria is the sole message promoting net use, little remains to encourage individuals to use their nets in a context of reduced malaria incidence. There is a considerable gap in the evidence regarding appropriate and effective messaging during periods of low malaria risk. It is largely unclear how to motivate populations who perceive that malaria is no longer a major threat. This is an important issue to address as malaria prevention efforts approach the elimination of the disease in certain regions and countries.

**Figure 1:**
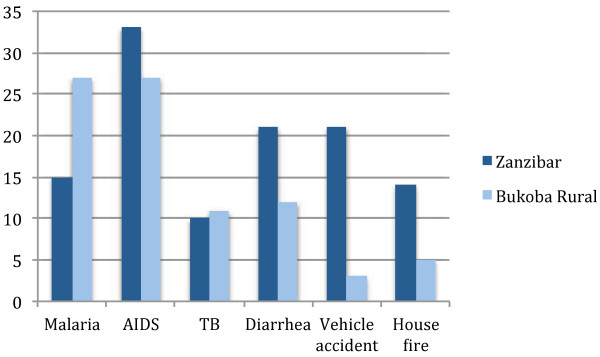
Summed rankings of diseases and calamities, by study site.

Elements of the Health Belief Model (HBM) [18,19] and the Theory of Reasoned Action (TRA) [20,21] were used to develop the conceptual model for this study (Figure 1) and the discussion guides. Beer et al. demonstrate effectively that HBM can be used to interpret net use behaviours in the context of reduced risk in Zanzibar [17]. Constructs from the HBM included perception of risk (composed of perceived susceptibility and perceived severity of illness), self-efficacy, perceived benefits, perceived barriers, and cues to action (such as presence of mosquitoes). According to the HBM, an individual will take preventive action if they believe that action will prevent illness and if they have the desire to avoid that illness. The TRA states that individuals make decisions based on subjective norms, attitudes and beliefs about the outcome of an action, which lead to intention to perform behaviour and subsequently the behaviour itself.

This article expands on the recent studies that explored perceptions of malaria and bed net use after a reduction in malaria incidence during interviews conducted in Zanzibar in 2007 and 2012, respectively [17,22], by focusing on how non-malaria benefits of nets are perceived by those living in areas of reduced malaria transmission, and how these findings inform message development for behaviour change communication interventions.

## Methods

### Study population

This research was comprised of 12 focus group discussions (FGD) with purposively sampled participants in Bukoba Rural and Zanzibar. However, one of the FGD recordings had technical problems and could not be recovered. Therefore, the results presented report on data gleaned from 11 FGDs. In each of the two sites, participants were purposively sampled by age, sex and net user status. Each site had focus groups with men aged 18–49, women aged 18–49, and women over the age of 50 years. Older women were selected as a separate focus group distinction since they would be better able to talk about changes in malaria risk, net availability and net use over time. All study participants were residents of Bukoba Rural or Zanzibar. Focus group sizes ranged from seven to 11 participants and a total of 35 men and 60 women participated in the FGDs.

### Study sites

Bukoba Rural and Zanzibar were chosen as study sites due to their significant malaria efforts, in particular the mass net distribution and indoor residual spraying campaigns over the past six years. For the four months prior to fieldwork, Bukoba Rural had its universal coverage campaign and subsequent Hang Up visits to provide nets and promote their use. In both sites, heavy rains fall from March to May and short rains from September to November in Zanzibar (October to December in Bukoba); hot season occurs in December (Zanzibar), January and February (Bukoba). In Kagera, three wards within Bukoba Rural District were selected (Kikomero, Rutete and Butelankuzi); in Zanzibar, two shehia in Zanzibar Urban District (Mkele, classified as rural, and Mlandege, an urban site) were selected.

### Procedures

Data was collected from 29 November to 6 December, 2011 by a team of four data collectors trained on study design, aims of the study and ethical treatment of participants, using pretested, semi structured interview guides. In Bukoba Rural, the District Malaria Focal Person, village chairpersons and community volunteers conducting Net Hang-Up visits worked with the study team to identify households that owned at least one net and used, or did not use nets. In Zanzibar, the Zanzibar malaria control programme official plus the Sheha (lowest government official) identified households. Potential participants were screened using a recruitment/screening tool. Interviews were conducted in Swahili. Interviewers were trained to distinguish between malaria fever (homa ya malaria) and non-malaria fever (homa isiyokuwa ya malaria) as Kiswahili words for fever and for malaria have been previously shown to be context-dependent [10]. Transcripts confirmed that participants made this distinction. Discussions touched on other types of fever (homa) but these findings relate to homa ya malaria.

### Data analysis

FGDs were audio recorded, transcribed verbatim, and translated into English. Transcripts were coded in Atlas.ti version 6.2 software using a hybrid approach of inductive and deductive coding. One individual coded all of the transcripts and an analysis team assessed outcomes of interest by region, sex, and user status.

### Ethical considerations

Ethical approval for this study was obtained from both the Johns Hopkins University School of Public Health Institutional Review Board in Baltimore, MD, USA and the University of Dar es Salaam Directorate of Research and Publications. Participants provided oral consent prior to participating in the study. No personal identifiers were recorded or transcribed.

## Results

### Perceived risk of malaria

As described in Witte’s Extended Parallel Process Model [23] and in the HBM [19], perceived risk is composed of both the perceived severity of the risk and the perceived susceptibility to the risk. Perceived severity of the threat is the danger that malaria poses; perceived susceptibility is informed by the perceived probability of getting malaria, or developing severe malaria. Participants spoke about changes in both severity of the risk of the malaria as well as their own susceptibility.

To first situate perceived susceptibility of malaria compared to other diseases or calamities, participants were asked to sort various diseases (malaria, HIV, diarrhoea, tuberculosis) known to be important health problems [24] or other calamities (house fire, vehicle accidents) as having either a ‘high chance’ of happening or a ‘low chance’ of happening. Participants in Bukoba Rural tended to agree that malaria and HIV had a high chance of occurring. Tuberculosis, diarrhoea, vehicle accidents and house fires were generally rated as ‘low chance’ or ‘less likely’ to occur. In Zanzibar, however, malaria was rated as ‘low chance’ by two of the five groups. HIV remained a high chance occurrence, along with vehicle accidents.

“Malaria according to what I think it has a high chance because despite the fact that there is no severe malaria but every day you will hear this one has malaria and this one has malaria; so in short I think that it has a high chance of happening”. (Older female, Zanzibar)

Participants were asked to rank the diseases or calamities rated as ‘high chance’ of occurrence. Participants interpreted this to mean a ranking of most worrisome to occur to the least worrisome. Scores of 6 (most worrisome) through 1 (least worrisome) were assigned to the rankings, and to account for the ‘low chance’ ratings, these items were given a −1 score. Malaria and HIV were perceived as the most worrisome, as shown in Figure 1. Zanzibari participants rated malaria lower than HIV and vehicle accidents, citing reductions in malaria.

### Reduction in overall risk

To understand the respondents’ perceptions of risk both in the past and at the time of the interview, discussion groups were asked about how fevers “nowadays” compared to “in the past’. Only a few participants felt that malaria had in fact increased or become more severe over recent years. These were mainly older men or women, who felt that this increase was due to a loss of earlier immunity, gained through diet or traditional medications. Another noted that while the occurrence of malaria may not have increased, the disease itself had become more severe. However, the few examples of increased sense of risk were however greatly outweighed by the number of responses expressing an overall reduction in perceived malaria risk. Many respondents specifically noted a reduction in severe malaria or convulsions (degedege in Swahili) as evidence for the decline. Respondents cited the availability of LLINs, indoor spraying, and quality drugs at the health facilities, behaviour change communication, and sanitation improvements as the primary reasons for the decline in both severe and non-severe malaria.

R: “To say the truth I see malaria has decreased because I am talking about a ward in my village and I am a secretary of a women’s group. Most of times there were many children’s deaths during rainfall but this time they are few.” (Older female, Bukoba Rural)

R: “We fear it but not like in the past”.

I: What are reasons behind that?

R: “We sleep under nets everyday and there are drugs to treat the disease. If you are sick and you go to a dispensary you’ll be given treatment like Alu [artemether-lumefantrin] tabs, septrin, paracetamol and if it’s a child s/he will be given quinine injections and then s/he may recover”. (Female, Bukoba Rural)

R1: “It is not the same as in the past, people didn’t bother about malaria in the past but now they are using mosquito nets properly because they want to prevent themselves from getting sick. They have changed.

R6: They have changed because in the past we used local herbs when we suffered from malaria…now we go to hospital for treatment”. (Female, Bukoba Rural)

Health education was cited as playing a part in the change in risk. Participants noted that in the past, people often went first to traditional healers, and then to the hospital when those treatments did not work. Now, “everyone understands” that they should go to the hospital quickly.

R: “Because in the past people did not understand the need of rushing to the hospital and many did not know that diseases could be treated at the hospital. Some were preventing others to go to hospital because they believed that children might die after being injected or given oral medicines. But as we are being educated then we came to know that when a child develops convulsions and hurried to a hospital, she gets well quickly after being infused with some medicines. Therefore right now many people have understood that convulsions are coming from malaria. In the past we used to call it Ezabo in Haya language, we did not know it was malaria”. (Older female, Bukoba Rural)

R: “The danger was significant because we had no knowledge and few people used nets and when they didn’t go to hospital when someone got sick, they used local herbs such as neem and others. Now when someone gets sick they rush to hospital, Alhamdulillah, he gets treatment and recovers”. (Female, Zanzibar)

### Nets cannot prevent all bites

Response efficacy, or the respondents’ perceived efficacy of the LLIN at preventing malaria, was reduced or limited in several circumstances. Staying outside during the evening, or needing to travel or work and not having a net were recognized as potential opportunities to be bitten by mosquitoes and develop the disease. “There are many that are using bed nets but they still suffer from malaria”, noted one participant (older female, Bukoba Rural). Others echoed those sentiments, noting that some people never used nets but never fell ill, while others used nets and continued to get sick. Respondents also noted that the insecticide wears out, citing a need for new nets or for the retreatment kits (Ngao) that had been socially marketed for several years prior.

R: “[Malaria] is not preventable, you may find that at my place I am using a bed net but when I am travelling using a boat or bus the mosquitoes are biting me and I do not have protection so I will get malaria”. (Older female, Bukoba Rural)

R3: “I think that it has decreased a bit, malaria has decreased. Everyone has their own understanding, I can say that malaria has decreased and I keep on protecting myself, do you understand me, because if this mosquito has died that was born yesterday, the day after tomorrow there comes another one…because of this issue with the mosquito I don’t think if malaria will be eradicated. What you can only do is to protect yourself from them but saying that they will leave completely - they will not leave, because when they die there are others that are born, and then they die, and then new ones are born”. (Female, Zanzibar)

### Perception of risk complicated by rapid diagnostic tests

In the Zanzibar focus groups, the participants discussed the confusion arising from malaria testing, and the uncertainty of whether or not they had malaria and how it should be treated. Rapid diagnostic tests are widely available and used in both public and private clinics throughout Tanzania. Respondents expressed impatience with doctors who told them they did not have malaria, when the participants felt that they did. In some cases the participants sought out malaria treatment despite the doctor’s advice; in other cases, the doctors themselves provided malaria treatment even when malaria test results were negative.

R: “I would like to contribute there that when you go to the hospital, all the hospitals private ones and government ones you are told that you do not have malaria when you are tested for malaria but I am surprised that some of the doses are for malaria”.

I: Which doses?

R: “If you will be given, first of all now there are combination medications for malaria and it has a name written in green I do not know what, when you go there you are given while you do not have malaria and those medication for malaria despite the fact that you have been tested and they said you do not have malaria they still give you those medications for malaria”. (Female, Zanzibar)

Repeated negative test results contributed to a reduced perceived need for malaria preventive behaviours.

“I think now the usage of nets have decreased because there are few mosquitoes and when people go to hospitals to test for malaria, they are always found negative. When you feel joint pains, headache or fevers and then you go for a malaria test, the results are always negative though they'll give you anti-malarials, so because of this the usage of nets has decreased”. (Male, Zanzibar)

### Seasonal variations in perceived risk

In addition to the distinctions made between risk of malaria over the past few years, participants noted that malaria risk continues to change throughout the year. This has been more fully explored by Winch [10] but the link between seasons, weather and risk of malaria and anaemia remains strong according to some of the participants, particularly the older men and women. In addition, participants generally linked an increase in mosquitoes to an increase in malaria. November to December and June to July were months in which “mosquitoes are many” (Older females, Bukoba Rural).

In both study sites, but particularly in Bukoba, several respondents noted that hot seasons were in fact the time of highest malaria risk. Respondents attributed malaria to the hot weather and to wind, stating that mosquitoes bred during the rainy season and then commenced biting or “woke up” once it became sunny and dry. In Bukoba, respondents noted that they were “more afraid” of malaria during the months of May to August, the “sunny season” that follows the rainy season; “in June children usually fall sick and you find many sick children when you go to hospital” (Older female, Bukoba Rural). The perception that malaria risk was highest during the rainy season was more common in Zanzibar.

### Benefits of net use

While respondents agreed that malaria prevention was one of the main reasons for using nets, many also stated that there was no reason to stop using them just because the risk of malaria had apparently decreased. Rather, participants expressed that they were more motivated to use nets, in order to maintain its protective benefit from malaria, but also because they now found that LLIN use had become a habit, and that sleeping without a net contributed to worries and to a sense of discomfort. Respondents also stated that IRS campaigns did not reduce their need to use nets; behaviour change communication (BCC) campaigns had instructed them to keep on using the nets after spraying, and that the dual protection provided by the spray and the net were seen as complementary rather than duplicative.

R: “When you use mosquito net then the possibility of being bitten by mosquitoes is small so as the chance of developing malaria. But if you don’t use a net then the possibility is big”. (Male, Zanzibar)

Respondents mentioned the reduction in cases of malaria as both motivating and demotivating for net use. Some mentioned that it encouraged net use because community members had seen how well nets worked at preventing malaria and continued using them to remain safe from the disease. Other respondents indicated that the decrease in malaria prevalence had decreased net use in the community, because people no longer thought that malaria was a problem. This issue was even debated within the same focus group discussions.

R1: “I think now the usage of nets have decreased because there are few mosquitoes and when people go to hospitals to test for malaria, they are always found negative. When you feel joint pains, headache or fevers and then you go for a malaria test, the results are always negative though they'll give you anti malarials, so because of this the usage of nets has decreased”.

R2: “Also not all people stopped using nets. Others after seeing that malaria cases have decreased, they have increased the frequencies of sleeping under a net so as to avoid infections completely. Also in my opinion I think we should continue to use the nets more and or spraying the insecticides more so as to kick malaria away”. (Males, Zanzibar)

### Comfort and discomfort of nets

The non-malaria-related benefits of net use focused on the comfort of net use; these were the more salient subjective factors mentioned by respondents. Respondents indicated that increased comfort when sleeping under nets was a motivating factor for net use. Nets were said to prevent nuisance biting from insects and pests, provide warmth and contribute to getting a good night’s sleep.

The most commonly mentioned benefit of net use and IRS was the prevention of bites and interactions with pests in the household. Next to considerations of the prevention of malaria and illness, comfortable sleep was the most commonly cited benefit of net use. Ticks, mosquitoes, fleas, lice and bedbugs were very commonly mentioned pests that could be avoided. However, respondents also noted the protective effect of nets against other pests such as cockroaches, snakes, rats, centipedes, ants, houseflies, lizards, spiders, and even cats.

R:” It is not just for the mosquitoes, there are spiders, there are insects of every kind that will always be there- every kind of insect. Even that cat may be looking for rats but when it enters inside the room, the cat cannot pass there to go catch a rat. It has to go to the other side. If you are not sleeping inside a bed net, the cat will scratch you”. (Female, Zanzibar)

Many respondents indicated that this prevention of nuisance biting and pests was instrumental in helping them to achieve a good night’s sleep, or usingizi mwororo. They defined a good night’s sleep as sleep without disturbance. Many respondents linked the deep sleep to feeling healthy, energized and well-rested during the following day. As a respondent noted, “It helps people to have good health and when they wake up, they don’t feel exhausted.” (Female, Bukoba Rural). Most, but not all, respondents in both sites indicated that a mosquito net is a factor that can help them to get good, deep sleep. They expressed that the net keeps mosquitoes and other pests away from them while sleeping and allows them to sleep soundly.

R: “For those who are using the mosquito nets, it is true that it helps you to have a good sleep. For most people, when a mosquito bites you, the sleep disappears. When you are sleeping under a net, you get good sleep and everything is fine”. (Male, Bukoba Rural)

I: “What do you mean when you say ‘usingizi murua, mnono or mwanana’

R9: The kind of sleep with no worries

I: What do you mean?

R: To sleep without being disturbed by mosquitoes or bedbugs.

I: And how can a bed net cause you to have ‘usingizi mwororo’?

R8: Because the bed net finishes all the insects so you sleep luxuriously”.

(Male, Bukoba Rural)

A good night’s sleep was also characterized by a lack of worry about getting bitten by mosquitoes and developing malaria. Not only are individuals able to sleep soundly without the nuisance of being bitten, but they are freed from the fear of what consequences the bites might have.

R: “Be it the dry or rainy season when mosquitoes enter the house I was not worried that they might bite me because I was sleeping under the net”. (Male, Bukoba Rural)

R: “They like to sleep inside the bed net because you do not have to worry about getting sick”. (Male, Bukoba Rural)

One individual who did not like nets acknowledged that she could not sleep without one. The discomfort of mosquitoes outweighed the discomfort of feeling hot.

R: “I do not like it even a little bit but there are mosquitoes making it impossible to sleep without a bed net.

I: Why don’t you like it mama?

R: I was not used to it and it is hot sleeping in it”. (Older female, Zanzibar)

### Net use as a habit

Some respondents discussed the importance of continuous use of nets to maintain good sleep. They mentioned becoming accustomed to the net and the challenges of sleeping without one.

R: “When you are used to sleeping under a mosquito net, then that comfort would cease the moment that you stop using it because you will be disturbed by mosquitoes. Once you are used to it, then you can’t stop using the net.” (Male, Zanzibar)

R: “For me I see a mosquito net as a blanket and I am used to it”. (Older female, Bukoba Rural)

Respondents in both sites talked about developing a habit of consistent net use. When asked their reasons for using nets, they simply mentioned that they were “used to it.” While it was not completely clear how this habit was formed, in many cases it seemed to be a result of the positive outcomes associated with undisturbed sleep. In developing this habitual net use, they have become accustomed to nets and consider it a necessary part of life. Building this culture of net use is ideal for maintaining consistent net use throughout fluctuations in perceived risk.

“Though malaria has greatly decreased, there are insects which affect our sleep like cockroaches and non-malarial mosquitoes. We have to put a mosquito net and when you are used to it, then you can’t sleep without it because you will definitely feel naked”. (Male, Zanzibar)

R: They are still using the mosquito nets because of being used to them. I am already used to it. We started to use mosquito nets because of protecting ourselves against mosquitoes and therefore we are already used to them”. (Older female, Bukoba rural)

R: Children and adults, all of them [are using bed nets] and without a bed net you cannot sleep. Mosquitoes are still there, now as they are saying that malaria is not present but mosquitoes are still there and without a bed net you cannot sleep and your children you cannot put him/her without a bed net, it is a must that your child gets a bed net and even you will not be able to sleep without a bed net”. (Older female, Zanzibar)

### Advice for friends

In order to understand the words and arguments that Tanzanian individuals would use to convince others to use nets, the interviewers asked respondents what they would say to a friend that did not sleep under a mosquito net. Respondents reiterated benefits regarding malaria prevention, comfort and getting good sleep as persuasive arguments.

“I will convince my friend to use a mosquito net by telling her the effects of malaria and the benefits of using mosquito nets… The effects of malaria are recurrent sufferings, deaths and then when you go to hospital you use money and your activities become affected because when you suffer from malaria, you cannot do any work. The benefit of mosquito nets is that when you sleep under it you get a good and deep sleep, you don’t contract malaria, you don’t get bitten by mosquitoes and snakes or rats won’t fall down on you. Those are the benefits”. (Female, Bukoba Rural)

“For example if I know that you don’t use a net I’ll come to you straight and I’ll ask you if you are using a net. If you say no then I’ll ask you if you have one and if you have one then I’ll ask you why don’t you use the net… you will give me your reasons but I’ll ask you again during the times when you are not sleeping under a net do you sleep well… Even if you sleep well I’ll ask you again when was the last time you were sick… If you reply then I’ll advise you to use a net because there a lot of mosquitoes and you shouldn’t be satisfied because of the indoor residual spraying or other insecticides you use because not all insects die during the process so when you sleep they might bite you spreading the infection thus using a net helps you in avoiding them”. (Male, Zanzibar).

Some participants suggested that they would advise their friends to acquire a net or assist them with learning how to hang or use it. The various arguments presented by the respondents included considerations about knowledge promotion, net acquisition, sleeping well at night, malaria prevention and nuisance insect prevention.

### Barriers to net use

To facilitate analysis, barriers to net use were coded as ‘subjective’ or ‘objective’. Subjective barriers were primarily attitudinal. Objective barriers were primarily structural, such as someone being away from home or if the net was not available.

Subjective barriers to net use were mainly related to discomfort, either due to heat or insecticide. Participants noted that nets made them feel ‘squeezed’, ‘uncomfortable’, ‘hot’, and ‘itchy’. A small number of participants, mainly older women, complained that LLINs made it difficult to breathe. Hot weather exacerbated discomfort. Several participants also noted the challenges of waking up in the night and untucking the net in order to go urinate, seen as particularly challenging for elderly and pregnant populations. Participants also cited fear of burning nets with oil lamps or candles, the difficulty of using a net with grass mats, and not having enough nets for all members of the family, particular older children of different sexes who could not share the same sleeping space. Men in Bukoba Rural described a rumour of fertility problems caused by the insecticide from IRS and/or nets.

Objective barriers included being away from home, and having a net still damp from recent washing. Funerals presented a particular challenge for net use. Respondents indicated that they travel to participate in funerals, sleeping outside in their clothes, with just a blanket or sheet to cover themselves. Respondents noted the logistical challenges of attaching the net in an open area with no beds or mattresses, amidst an outdoor crowd, while others said it was common for people to stay awake all night. Others stated that it would be inappropriate to use a net at a funeral, since others would remain unprotected. Both men and women recognized the danger that lack of net use at funerals presented and noted the large crowds and the opportunities for transmission.

R: “For example it happens that you have a funeral at your house and you have nets just enough for your family then it is inhuman to sleep under a net leaving the guests sleeping outside without nets”. (Male, Zanzibar)

R: “We do not use nets at all in that occasion. When we go to funerals, we sleep outside and a bit fire is lit. Would you fix a net there? If there are any mosquitoes, then they will do their job perfectly”. (Male, Bukoba Rural)

“[At] that wake (mkesha) people will be dancing they will not be covering themselves with bed nets”. (Female, Zanzibar)

### Alternative uses of nets

As risk of malaria decreases it is possible that nets may be perceived as more useful for alternative uses than for malaria protection. A small number of people in both study sites discussed the repurposing of nets in their community for agriculture rather than for malaria prevention. Alternative uses of nets included protecting gardens, enclosing poultry, fishing, and collecting flying ants. Respondents noted that older, torn nets were primarily the ones that were repurposed. Only one of the 95 participants described the repurposing of a new net for an alternative use. In both Bukoba Rural and Zanzibar community leaders and Shehas had sanctioned those who misused nets by imposing fines or other punishments.

R: “When a mosquito net is torn or used up…my fellows usually use the torn one to make chicken huts by placing them on top. (Older female, Bukoba Rural)

R: “Others use the nets for fencing paddy fields but some use them in vegetable gardens…while I have already reported to the Sheha that, this net of mine has already been worn out. In that case I will give you a new one and the old one I use for fencing my vegetables.

I: What would the Sheha say if he finds out you have used the net for fencing?

R: Ahaha he knows that the net had already worn out.

I: Won’t he take any measures because you are using the net inappropriately?

R: it is because the net was worn out”. (Female, Zanzibar)

## Discussion

**Figure 2:**
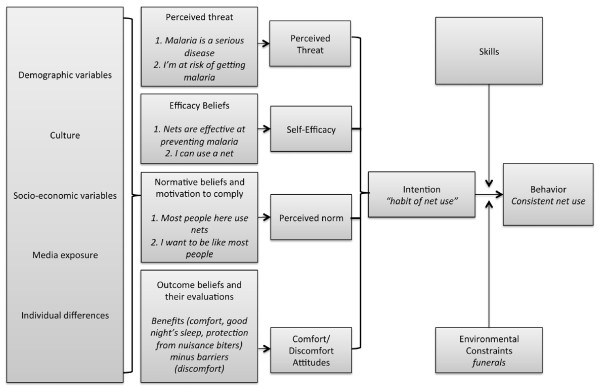
**Conceptual model for messaging in reduced transmission zones – adapted from the integrative model of behavioural prediction of Fishbein and Yzer **[26]**.**

The conceptual model below (Figure 2) is adapted from the Integrative Model of Behavioural Prediction (IMBP) [25], which combines variables of two well-known theories: the HBM [18,19], and the TRA [20,21]. The IMBP has the advantage of including intention to act, linked here with developing a habit of net use. The TRA and IMBP include normative beliefs (subjective norms and motivation to comply), which remain in this model but did not appear to play a significant role in respondents’ reasons for net use. Theoretical models are useful to inform message conceptualization, as messages that target the most important determinants of behaviour are more likely to be effective [26].

To summarize, the model, demographic variables, culture, socio-economic variables, media exposure, and individual differences play a role in individuals’ perceived threat, efficacy beliefs, normative beliefs, and outcome beliefs. Perceived threat, efficacy, norms, and outcome beliefs (here relating primarily to elements of comfort and discomfort) combine to influence intention to act, or the habit of net use. Intention is modified by skills (a relatively minor element, related to hanging nets) and by environmental constraints such as funerals or other structural barriers; finally leading to the behaviour itself, consistent or habitual net use.

Results from this study show that perceived risk of malaria has decreased among the respondents, particularly in Zanzibar, and that malaria control interventions are credited for the decline. In Zanzibar these findings confirm results from two recently published papers on perceived malaria risk [17,22]. Participants cited a reduction in the severity of malaria, that is, the number and frequency of severe cases or deaths from malaria in their communities and families, as well as a reduction in their perceived susceptibility to malaria. Decreases in both severity and susceptibility were attributed to the availability of LLINs and various drugs at health facilities, particularly Alu, as well to the IRS campaigns, improvements in sanitation and standing water, and improved education on the part of the population about malaria and its prevention.

Respondents expressed a sense of control over malaria “nowadays” as compared to “in the past”, again citing the availability of drugs and LLINs. The perceived response efficacy of these malaria control interventions was quite high; respondents agreed that nets in particular worked well at reducing mosquito bites, although several noted that there are still moments in the evenings or while travelling that net use is not possible and mosquitoes remain a threat. Respondents also felt confident that malaria drugs, in particular the first line ACT, artemether-lumefantrine or Alu, would be available at health centres and that they could access them there. This combination of perceived response efficacy, or belief in the effectiveness of the tool, and respondents’ own perceived self-efficacy at using nets, accepting IRS, and accessing drugs fits with the HBM where these two elements are the components of perceived self-efficacy.

Receiving negative diagnostic tests multiple times is likely to contribute to the sense of decreased risk of malaria. Willingness to trust the results of diagnostic tests is an important concern as malaria control efforts are scaled up, and represent a concrete example of the challenges that can arise when personal risk assessment is corroborated or overturned. Receiving a negative test result when symptoms indicate malaria also leads to cognitive dissonance for both providers and for patients or caretakers. One way that people resolve cognitive dissonance is to discount the effectiveness of the test itself, and as seen above, it appears common that patients continue to ask for malaria treatment and even doctors sometimes provide it despite the negative test results. In the absence of clear protocols or additional diagnostics for other causes of fever, this confusion is likely to remain a problem, even in environments like Zanzibar where parasitaemia rates among under five-year-olds are less than 1%. In addition, unnecessary treatment of suspected malaria is an inefficient use of resources.

Barriers to net use were in line with a recent review of reasons for not using nets [16,17]; discomfort from heat or feeling closed in were the most cited reasons. While wind-tunnel testing has shown that it is not in fact hotter or more humid inside a net, decreases in airflow do occur and can increase discomfort [27]. Perceived discomfort caused by insecticide, fear of the net catching fire, and the annoyance of getting in and out of the net during the night were the other most commonly cited reasons for not using a net. Older male respondents in Bukoba Rural expressed the fear of insecticide causing infertility or impotence; similar rumours were reported in Mara and Mwanza regions related to the IRS campaign [28].

The role of comfort in net use appears to be important among respondents in this study. Aside from malaria prevention, the main reason for sleeping under nets was because they provided comfortable sleep, and this was a key factor in consistent use of nets. Nets prevent nuisance biting from mosquitoes and other pests, and provide warmth during cold season. Respondents repeatedly expressed that they had become used to nets or were in the habit of using them. Programme planners speak of building a ‘net culture’, although this concept remains ill defined. A majority of people who believe that they cannot sleep without a net is almost certainly a key component of a net culture. Building habits of consistent net use despite variations in temperature and mosquito density is possible, as has been seen here.

Recent research has tended to focus on identifying the barriers to net use [16,17] or the malaria-related benefits of net use [22], perhaps assuming that malaria prevention is reason is enough. These findings indicate that non-malaria benefits do exist and may be exploited for developing messages to improve consistent net use.

### Recommendations for messaging as malaria risk declines

The interplay of perceived risk and comfort modulate net use behaviours in the study areas. Messaging to promote net use in areas like Bukoba Rural and Zanzibar should focus on highlighting these non-malaria benefits of net use as well as the preventive benefits; making an LLIN an essential component of a ‘good night’s sleep’ rather than simply a tool to prevent malaria may further entrench Tanzania’s net culture. Messages should also promote nets’ ability to protect the user from a wide variety of nuisance biting insects and pests. A good night’s sleep message can be extended further: a good night’s sleep enables a productive day at work or school, and ensuring good sleep over the long term could improve overall performance of a student at school or facilitate adults’ ability to perform their jobs well and provide for their families. Preferred channels of communication were not addressed in this study, but might include radio, health committee talks, schools, and mosques as described in Bauch et al. [22].

Perception of risk is complex and multifaceted, comprised of individuals’ assessments of their susceptibility, the severity of the disease, and how effectively the individual feels they can access prevention or treatment options. Focusing messaging solely on aspects of risk may be difficult to do in a way that resonates with a large number of people, as risk is too idiosyncratic to be targeted with a broad message, and concentrating BCC around malaria risk is likely to induce message fatigue. Those individuals who do not feel themselves at risk, or do not feel they have the means to manage the risk, are more likely to reject BCC messages on risk, as described in Witte’s Extended Parallel Process Model [23]. Messages that highlight the comfort aspects of nets and net use, however, have the potential to improve consistent net use among those who already believe that nets provide a degree of comfort, and provide a stronger rationale for those who are currently weighing the discomfort of net use from hotness or claustrophobia against the benefits of sound sleep and freedom from worries about malaria.

While the findings from this study are not generalizable due to the qualitative nature and the specific environments of the study sites, it is nonetheless important to consider that these types of non-malaria benefits of net use are likely to be important for net use in other countries or regions where malaria transmission is falling and LLINs remain important tools to maintain those gains. Messages may work to bolster net use in low-transmission or low-risk seasons such as the dry season or hot season, even in areas where malaria transmission has not yet been noticeably reduced. Findings from Bukoba Rural and Zanzibar may provide clues for other countries or regions that are facing the challenge of maintaining protective behaviours as malaria transmission and the threat of severe malaria fall. Zanzibar is in a pre-elimination phase while Kagera remains in a control phase, and both regions may stay in these phases for decades or more before being able to progress to the next phase [29], assuming sufficient funding is made available. Continued use of LLINs will be a crucial tool in making progress towards these goals. Targeted messages that support consistent net use, for populations in reduced-transmission zones, must be identified in order to support malaria prevention efforts.

### Limitations

The data collected were qualitative in nature and as such, the specific findings cannot be generalized to other contexts. However, the data does provide programme implementers with a framework for considering risk/benefit calculations related to net use. Secondly, while the study was initially designed to enable a doer/non-doer analysis, these groups were actually less distinct than initially thought. Many of the respondents’ net use statuses fluctuated seasonally and based on the availability of nets. Therefore, the doer/non-doer analysis was not a useful distinction.

This study also had some logistical challenges that may have affected the results. Zanzibar participants were from two shehia in a single (large) district, Urban West. One FGD had technical problems and was not recovered nor included in this analysis. While saturation appeared to be reached within the other 11 focus groups, it is possible that the remaining group of older Zanzibari women or participants from other parts of the island would have provided additional perspective.

## Conclusion

Results showed that perceived risk of malaria has decreased among the respondents, and that malaria control interventions are credited for the decline. Participants cited reductions in both the severity of malaria and in their perceived susceptibility to malaria. However, malaria was still considered a significant threat. Participants’ conceptualization of risk appeared to be an important consideration for net use; net use was cited as a way to reduce one’s personal risk of malaria as well as a major contributor to the overall reduction of malaria in both regions. At the same time, comfort and aspects of comfort (getting a good night’s sleep, avoiding nuisance biters) appeared to play a large role in personal decisions to use nets consistently or not. Barriers to comfort (feeling uncomfortable or trapped; perceived difficulty breathing, or itching/rashes) were frequently cited as reasons not to use a net consistently. While it was apparent that participants acknowledged the malaria-prevention benefits of net use, the exploration of the risk and comfort determinants of net use provides a richer understanding of net use behaviours, particularly in a setting where transmission has fallen and yet consistent net use is still crucial to maintaining those gains. Future behaviour change communication campaigns should capitalize on the non-malaria benefits of net use that provide a long-term rationale for consistent use even when the immediate threat of malaria transmission has been reduced.

## Competing interests

The authors declare they have no competing interests.

## Authors’ contributions

HK designed the study and interview guides, analysed the data, and drafted the manuscript; DL contributed to the interview guides, conducted analysis, and drafted portions of the manuscript; DR contributed to study design, and interview guides, supervised data collection, data processing and preliminary data analysis. AA facilitated fieldwork in Zanzibar and provided perspective on the analysis. All authors read and approved the final manuscript.
